# Wrong schools or wrong students? The potential role of medical education in regional imbalances of the health workforce in the United Republic of Tanzania

**DOI:** 10.1186/1478-4491-8-3

**Published:** 2010-02-26

**Authors:** Beatus K Leon, Julie Riise Kolstad

**Affiliations:** 1 Centre for Educational Development in Health, Arusha, the United Republic of Tanzania; 2 Chr Michelsen Institute & Department of Economics, University of Bergen, Norway

## Abstract

**Background:**

The United Republic of Tanzania, like many other countries in sub-Saharan Africa, faces a human resources crisis in its health sector, with a small and inequitably distributed health workforce. Rural areas and other poor regions are characterised by a high burden of disease compared to other regions of the country. At the same time, these areas are poorly supplied with human resources compared to urban areas, a reflection of the situation in the whole of Sub-Saharan Africa, where 1.3% of the world's health workforce shoulders 25% of the world's burden of disease. Medical schools select candidates for training and form these candidates' professional morale. It is therefore likely that medical schools can play an important role in the problem of geographical imbalance of doctors in the United Republic of Tanzania.

**Methods:**

This paper reviews available research evidence that links medical students' characteristics with human resource imbalances and the contribution of medical schools in perpetuating an inequitable distribution of the health workforce.

Existing literature on the determinants of the geographical imbalance of clinicians, with a special focus on the role of medical schools, is reviewed. In addition, structured questionnaires collecting data on demographics, rural experience, working preferences and motivational aspects were administered to 130 fifth-year medical students at the medical faculties of MUCHS (University of Dar es Salaam), HKMU (Dar es Salaam) and KCMC (Tumaini University, Moshi campus) in the United Republic of Tanzania. The 130 students represented 95.6% of the Tanzanian finalists in 2005. Finally, we apply probit regressions in STATA to analyse the cross-sectional data coming from the aforementioned survey.

**Results:**

The lack of a primary interest in medicine among medical school entrants, biases in recruitment, the absence of rural related clinical curricula in medical schools, and a preference for specialisation not available in rural areas are among the main obstacles for building a motivated health workforce which can help correct the inequitable distribution of doctors in the United Republic of Tanzania.

**Conclusion:**

This study suggests that there is a need to re-examine medical school admission policies and practices.

## 1. Background

The United Republic of Tanzania, is among the many countries in sub-Saharan Africa facing a human resources crisis in its health sector, with a small and inequitably distributed health workforce [1] that shoulders a disproportionately high burden of disease[2]. Although all poor countries in the world face a severe human resource crisis in their health sectors [3,4], the problem is most acute in Sub-Saharan Africa, in which an estimated workforce of 750 000 health workers in the region serves 682 million people [2]. By comparison, the ratio is 10 to 15 times higher in developed countries. Moreover, this estimated workforce of doctors, nurses and allied health workers in Sub-Saharan Africa constitutes 1.3% of the world's health workforce, while Africa suffers from 25% of the world's burden of disease [2].

A minimum level of a health workforce of 2.5 health workers per 1000 people is required to achieve the Millennium Development Goals [5]. Africa is far from this level with a health workforce density that only averages 0.8 worker per 1000 people, while the world median density of health personnel is 5 per 1000 people [5].

There is a positive correlation between health worker density and various health indices, most notably infant mortality rate, maternal mortality rates, and various disease specific mortality and morbidity rates [6,7]. An increase in the number of health workers per capita is associated with a notable decline in the rates mentioned above. As a consequence, it has been argued that health worker shortages have impeded the implementation of development goals in many poor countries [8].

### The number and distribution of medical doctors in Tanzania

The United Republic of Tanzania has an active supply of 49 900 health workers, which translates into a staff-per-population ratio of 148 per 100 000 [9]. Other studies show that physicians (MD and above) account for 1% of the health workforce, keeping the physician-per-population ratio at 4.2 per 100 000 people ([10], [11]). In the WHO estimates of health personnel in 1998, the United Republic of Tanzania had the lowest ratio of qualified staff to population of all African countries [12]. The intermediate medical cadres (clinical officers and assistant medical officers) locally trained at a level below the medical degree constitute 14% of the workforce, and in some instances it is natural to include them into the physician group. This apparently improves the physician per 100 000 population ratio to 25.3 [10], which to a certain extent reflects the reality of rural health services in the United Republic of Tanzania, in which rural district hospitals have been primarily operated for many years by assistant medical officers and clinical officers [13].

The health workforce of the United Republic of Tanzania is very unevenly distributed between the rural and urban districts [1]. Although the Tanzanian rural population stands at 66% to 80% of the total population ([14,15]), only one-third of all doctors in the country work in rural areas [6]. This inequity has persisted despite an almost fivefold increase in the annual medical student intake in both public and private universities since 1997 [16].

### Medical students and the HRH imbalance

If we believe that preferences are important to health workers' choice of a job and job location, the preference for the place of practice necessarily plays a vital role in the distribution of human resources for health (HRH). Research evidence points at specific medical student characteristics that can predict practice preferences ([17]; [18]; [19]; [20]; [21]; [22]; [23]). An urban bias in the choice of practice place ultimately results in an inequitable distribution of human resources for health (given that we know that the present imbalance favours urban areas and that there is a high unmet demand for health workers in rural areas). Thus, in order to reduce the costs of evening out this current imbalance, it is important to examine which types of students and future clinicians are likely to prefer a rural practice - and why.

### Previously identified predictors of willingness for rural medical practice

In an extensive systematic review of factors associated with the recruitment and retention of primary care physicians in rural areas, Brooks et al. (2002) divided the factors considered into pre-medical school factors, medical school factors and residency training factors [24]. We will adopt a similar division for the types of predictors of the willingness of the medical finalists studied to choose a rural practise, focusing on background characteristics, motivation for medical studies and the influence of training institutions. As, at the time of writing, these students are not yet working out in the field, however, it is not possible to examine the residency training factors.

#### Rural background

In the United States of America, Rabinowitz et al. analysed more than 90 variables for 1609 Jefferson Medical College graduates over 20 classes [18]. 'Growing up in a rural area' turned out to be the most important independent predictor of practise in a rural area, and other studies support this finding. Brooks et al. (2002) identify rural origin as the variable most strongly correlated with recruitment to rural areas [24]. Doctors with a predominantly rural childhood are up to four times more likely to enter a rural practice than those growing up in urban areas [21]. In the same study, sub-predictors associated with a rural background such as having a rural primary school education increase the likelihood of rural practice. Having a family and living in a rural area has similarly been found to be positively associated with long-term plans to practice in such an area [21].

It is clear that human resources planning and policy have failed in several respects to deliver an appropriately trained workforce to the places where it is most needed [25], with one of these aspects the intake of students. An urban bias in the selection of candidates for training has been suggested as a failure on the part of human resources development policies in many countries. Research has shown that rural students face many particular barriers to pursuing medical education, as apart from geographical isolation, rural communities generally lack the facilities and resources to support their candidates for training [17]. Kamien (1987) addresses the issue of availability from a slightly different perspective and points to the fact that rural students often lack access to educational opportunities available in suburban settings. Students from rural schools are also less likely to perform well in their final high school examinations; hence, they are often unable to meet the entry requirements set by most universities, and are often not able to compete with their urban counterparts [26].

##### Motivation for medical studies

'Entering medical school with plans to become a family physician' is the second most important independent predictor of rural practice in the study by Rabinowitz et al. from 1999. Based on their extensive review, Brooks et al. (2002) similarly identify specialty preference as the factor most strongly correlated with recruitment to rural areas aside from having a rural background [24].

##### The influence of training institutions

In a survey of 189 medical students at Monash University in Australia, Somers (2000) finds that the intention to practice in rural areas increased among a group of students who were exposed to rural attachment and assigned a rural mentor. However, it was crucial to this programme that the rural attachment was considered a positive experience. A negative experience with rural attachment was worse than no attachment at all, and the same was reported with respect to having a rural mentor [19]. In another study, undergraduate and postgraduate clinical experience in a rural setting was found to be the second strongest predictor of rural practice [21]. A related finding to that of Somers was reported in Azer et al. (2001), in which the perception of the state of rural health services clearly influenced Australian medical students' choice of a rural career [20]. Such perceptions are likely to be influenced by the attitudes the students are met with at their training institutions, as well as by the personal experiences gained from fieldwork in rural settings.

It is important to note that factors concerning rural jobs directly, e.g. working conditions and future career prospects will also be important in the willingness to practice in rural areas. In the following, however, using a unique data set containing Tanzanian MD students' preferences for rural postings, we will address some of the issues concerning personal characteristics, intake to medical studies and the effect of training. We will particularly concentrate on what effect medical training has on the motivation of the future doctors of the United Republic of Tanzania, and analyse to what extent medical training influences their willingness to pursue a rural medical practice. To the best of our knowledge, this is the first quantitative study analysing Tanzanian doctors' preference for rural practice.

## Methods

### The data

A cross-sectional survey was conducted in 2005 among fifth year undergraduate medical students at the medical faculties of MUCHS (University of Dar es Salaam), HKMU (Dar es Salaam) and KCMC (Tumaini University, Moshi campus). At the time of this data collection, there were two more institutions educating medical doctors in the United Republic of Tanzania, although one of them was still in start-up phase and the other in the middle of an administrative crisis, so these institutions were left out of the sample. A structured questionnaire was administered to 130 fifth-year medical students, representing 95.6% of that particular cohort in these three universities. The choice of fifth year students was made based on the fact that they were in their final year of study, thus implying that they had already covered their community health rotation and should have had at least some exposure to essential community health issues. After more than four years in medical school, a medical student was further expected to have gathered adequate clinical knowledge and exposure to inform him/her in making the decision of where to seek employment and to have at least a rough idea about his/her intended further professional development.

While data stemming from surveys can be very informative and yield structured information pertaining to issues at the core of a research question, there are some possible problems with this type of data that need to be taken into careful consideration, particularly when applied to conducting quantitative analysis. Answers to a survey are likely to be biased towards socially acceptable views, and in our case, the data were collected in personal interviews in which the researcher filled in the questionnaire while sitting with the respondents. It seems reasonable to believe that in such a situation the respondents are prone to give answers which are biased towards their perception of the researcher's views on the topic being discussed. This does not mean, however, that the information obtained from these surveys is not valuable or cannot be trusted. On the contrary, we argue in our discussion that the survey results can reveal valuable insight into which factors are most important in regard to the willingness to work in rural areas.

### Model specifications

A standard probit analysis is applied in order to explore the relation among various background and training characteristics and the willingness to work in rural areas. The applied probit model derives the probability of individual *i *accepting a rural job, and this probability is denominated y_i_. The model also derives the relation between the probability of taking the rural job and various explanatory variables such as personal characteristics. The model is specified like this:

The dependent variable, *y*_*i*_, is binomial and takes the value 1 if respondent *i *answers that he/she would be willing to accept a posting in a rural area, and 0 if he/she does not accept. We have deliberately chosen the concept of *accepting *a rural job as the dependent variable. Since we already know that there is a problem in recruiting enough doctors to rural areas, it seems likely that there will be a very low rate of respondents answering that they will actively apply for such jobs. If we ultimately want to perform an analysis that can help in forming practical policies for recruiting more doctors to rural areas, it is important to also include those that may not actively seek a rural job, but who could be convinced that it is a real option after receiving a concrete offer.

On the right hand side of the equation there are four main groups of independent variables, namely personal characteristics like sex and age specified in the model by a vector *c*_*i*_, rural background specified by a vector *x*_*i*_, motivation factors specified by a vector *z*_*i*_, and the characteristics of the training specified by a vector *r*_*i*_. The coefficients of these vectors are specified as *β*_*i*_*, α*_*i*_*, δ*_*i*_, and *μ*_*i*_, respectively; and finally *ε*_*i *_is an iid-normally distributed error term which can be thought of as an unexplained residual. Descriptive statistics for the independent variables are provided in the next section.

The regression results are reported as marginal effects, as the coefficients in a probit model are not easily interpreted. Furthermore, the marginal effects give us the opportunity to compare the relative importance of the variables studied on the willingness of accepting a job in a rural area. The marginal effects are simply given by the expression, *∂ Prob(y = 1)/∂ c*, in the case of general background characteristics; *∂ Prob(y = 1)/∂ x*, in the case of rural background characteristics; *∂ Prob(y = 1)/∂ z*, in the case of motivation variables; and *∂ Prob(y = 1)/∂ r*, in the case of characteristics of the training.

## Results & Discussion

### Descriptive statistics

#### Intake and rural background

In an earlier application of the data set, Leon (2005) found that only 30% of the final year medical students in the sample had a rural background (grew up and spent most of their lifetime in a rural area). Another 35% were from Dar es Salaam, while the remaining 35% were from other urban areas in the United Republic of Tanzania [22].

As can be seen from Row 1 in Table 1, male students generally outnumber female students by almost twofold. Our data does not tell us whether females do not apply as often as males or if their grades are not good enough, although we assume that it is a mixture of both. In particular, women with dependants are underrepresented.

**Table 1:** Descriptive statistics

	Female	Male
Number	46	84
Average age	26.9	26.8
% with dependants	20	38
Grew up in rural area, %	20	36
Primary and/or secondary education in rural area %	26	50
Parents live in rural area %	17	36
Rural working experience before medical studies %	13	31
Rural fieldwork during medical studies %	89	81
Planned medical specialty %	67	63
Planned public health specialty %	22	21
Planned other specialty %	0	6

Note that the category 'Other' mainly represents business administration.

The proportion of students with some type of rural experience before medical studies is predominantly higher among male than among female students as we can see in rows 4-7 in Table 1.

These findings indicate how unlikely it is for rural students, especially girls, to pursue a medical education at a university. The fact that only 20% of the graduating females in medical schools have a rural background in a country where 80% of the population is rural depicts a sheer imbalance. The cohort represents more than 95% of the students who were enrolled in the course 5 years earlier, so the throughput has been good. We can therefore assume that this imbalance is not due to more dropouts among rural and female students, but is more likely that the problem can be traced back to pre-recruitment factors. Since all universities select only the best of the applicants for enrolment, this indicates that most rural students either do not qualify for admission, or are unable to compete with their urban counterparts.

#### Motivation

After five years in medical school, only 8% of the students report being more motivated for a medical career than they were upon entry. Two-thirds report feeling less motivated, and only 25% retain the initial level of motivation they had at the time of joining the medical school [22]. The implications of producing demotivated doctors in a country with a poor supply of doctors are potentially enormous, as it is likely that both the probability of leaving the health sector and delivering lower quality services are positively correlated to demotivation. If this is the case, valuable resources have gone to waste.

As we can see from the two first rows in Table 2, the share of female students who retained their initial level of motivation for a medical career is larger than the share of male students. Our data set does not allow us to investigate why this is so, but it has been well established that women sometimes have different preferences from men, see for instance [27,28]. Their motivation level may therefore be affected differently as a result of their training, even though they attended the same training programme. For more on gender HRH, see [29].

**Table 2:** Change in motivation according to sex and background

	% Less motivated	% No change in motivation	% More motivated
Female	59	33	9
Male	71	23	6
Dar es Salaam	58	33	9
Urban	69	28	3
Rural	74	17	9

Table 2 also shows that the share of students who are demotivated is higher among those with a rural background than among those with an urban background. Since previous evidence has indicated that rural students are more likely to take jobs in rural areas, this may actually represent an extra challenge for those recruiting doctors to these places.

In Table 3, students are grouped according to their initial motivation for studying medicine, and this motivation is shown for the different groups at the end of their studies. The groups reporting the most demotivation are those who decided on a career in medicine in anticipation of a better future, higher social status, guaranteed employment and monetary gain. Those who decided to attend medical school because they believed it was the best choice for using their high school education, and those who thought that a medical education would give them appreciation and respect, are also very demotivated by the end of their studies. The highest level of motivation is found among those who attended medical school, driven by a personal interest in medicine, regardless of whatever else that decision would bring. This picture fits relatively well with the reasons given for demotivation; it turns out that both poor financial remuneration and working environment were the most common reasons for being demotivated, as summarized below in Table 4:

**Table 3:** Change in motivation according to initial motivation for medical studies

Initial motivation	% Less motivated	% More motivated	% No change in motivation
A better future	100	0	0
Appreciation/Respect	100	0	0
Good highschool grades	100	0	0
Higher social status	100	0	0
Influence from relatives	100	0	0
No specific reason	100	0	0
Sure employment	100	0	0
To earn money	100	0	0
Prestige	78	0	22
Desire to be helpful	65	12	24
Parents persuasion	58	11	32
Personal interest	55	2	43
Peer influence	50	50	0
Relative is a doctor	0	0	100

**Table 4:** Reported reasons for demotivation

Reasons	% reporting this as reason no. 1
Doctor's salary too low	36
Low income	18
Poor working conditions	15
Heavy workload	9
Poor learning environment	6
Intimidation by lecturers	3
Course too long	2
Frustration from lecturers	2
Government too irresponsible	2
Not respected as a student	2
Tension at medical school	2
Doctor's poor life standard	1

#### Specialisation intentions

The medical students analysed in this study seem to be very intent on further education as we can see in the last three rows of Table 1. Only 11 out of 130 students reported that they are not intending to continue on to postgraduate studies. On average, students are willing to wait 2.1 years before going for further studies, but we do not know if they intend to practice medicine in the period between their studies.

The fact that the proportion of students intending to pursue clinical specialties is significantly high may not be good news for the Tanzanian health care system, where such specialists only work in regional and referral hospitals located in urban centres. The ultimate result of this trend is an urban bias, whether intended or not. Since most medical specialists primarily work in cities, an over-concentration of mono-specialty training continues to augment the imbalance in HRH distribution. Candidates for specialist training are normally derived from the pool of generalists. As general practice is not regarded as a specialty in the United Republic of Tanzania, the training of specialists reduces the number of general practitioners. It seems timely to ask to what extent specialist training is and should be need based. Maurice King described the doctor working in a rural district as "twenty surgeons in one" [30], referring to the multiple skills that this type of doctor needs in order to effectively deliver services in such resource-poor settings. Instead of "converting" doctors into mono-specialists and thus removing them from the district health system, it could be a solution to train them further as general practitioners, and give them the same remuneration and promotion possibilities that doctors with postgraduate qualifications receive. An alternative solution would be to start with specialist training in family or rural medicine.

#### Rural practice during the training

Most students had some exposure to rural areas during medical training as shown in Row 8 of Table 1. The few (20% males and 12% females) who lacked this exposure attribute it to a lack of funds to travel to and live in the rural areas during training, and this problem seems to be most common among privately sponsored students.

### Regression analysis of the willingness to accept a rural medical job after studies

#### Rural background

Several variables can indicate a rural background. We have applied three different ones:

a) the respondent has grown up and spent most of his/her childhood in a rural area (we have also included a dummy variable for growing up in an urban area other than Dar es Salaam, as a childhood in the capital is in many aspects very different from a childhood in other urban areas);

b) the respondent underwent primary and/or secondary education in a rural area; and

c) the respondent's parents are living in a rural area.

To recapitulate, the probability that a respondent is willing to accept a job in a rural area depends on some simple demographics, the three different indications on rural background, and an unexplained residual, *ε*. Results from this regression are presented in Table 5 under the column entitled "Model 1".

**Table 5:** Results from regressions

Variable	Model 1	Model 2	Model 3
Male student	0.116	0.142	0.153
	*(0.117)*	*(0.127)*	*(0.128)*
>26 years	0.305**	0.368***	0.329**
	*(0.143)*	*(0.146)*	*(0.154)*
Number of dependants	-0.045	-0.089	-0.087
	*(0.120)*	*(0.129)*	*(0.132)*
Rural background	-0.056	-0.105	-0.144
	*(0.174)*	*(0.191)*	*(0.203)*
Urban background (other than DSM)	0.245**	0.286***	0.284***
	*(0.110)*	*(0.112)*	*(0.112)*
Schooling in rural area	0.005	-0.080	-0.031
	*(0.123)*	*(0.133)*	*(0.145)*
Parents live in rural area	0.501***	0.557***	0.537***
	*(0.102)*	*(0.099)*	*(0.104)*
Motivated by interest in medicine		0.016	0.022
		*(0.122)*	*(0.122)*
Specialisation in medicine		-0.305	-0.299
		*(0.211)*	*(0.208)*
Specialisation in public health		-0.505**	-0.501*
		*(0.250)*	*(0.263)*
Community health service during studies			-0.342**
			*(0.163)*
Fieldwork during studies			0.285
			*(0.262)*
y = Pr (accept a job in a rural area)	0.614	0.616	0.629
Number of observations	106	106	106
LR chi2 (12)	24.91	28.47	31.28
Pseudo R^2^	0.174	0.199	0.219
Prob > Chi2	0.0008	0.0015	0.002

The coefficients reported marginal effects as described in Section 2. Standard errors are given in brackets.

The stars indicate the significance of the estimates (* 10% level, ** 5% level, *** 1% level).

It turns out to be a significant result that people over the age of 26 are more likely to accept a job in rural districts than younger persons. The likelihood of accepting a rural job increases by 30 percentage points when the age is above 26 (significance level of 5%), and this result is in line with the findings of McDonald et al. [21].

Our results further confirm another finding from previous research, namely that personal links to rural areas can be an important determining factor in the willingness to work in rural areas [23]. When the parents live in a rural district, the probability that their child will accept a job in a rural district rises by 50 percentage points, and this finding is significant at a level of 1%. Thus, family seems to be an important factor when young doctors are deciding where to work, although it is a bit unclear as to what the policy implications of this would be.

It is possibly more important for policy purposes to find out whether it is of concern that students with a rural background are accepted at medical schools. Somewhat surprisingly, students from an urban background other than Dar es Salaam are more likely to accept a job in a rural district (significant at a level of 5%) than the other respondents. However, we were not able to establish any significant relation between growing up in and accepting a job in a rural area. This may be due to a small sample size and multicollinearity (discussed below), or the characteristics of the sample in which students from Dar es Salaam are overrepresented and rural students underrepresented. Searching for intake strategies that allow more students from a background outside Dar es Salaam into the medical schools could, according to these results, form one way of increasing the general willingness of doctors to accept rural jobs.

#### Motivation for medical studies

We proceed by also including factors that were important for choosing a medical career. In the descriptive analysis, a personal interest in medicine was by far the most important motivational factor for studying medicine, which makes it a natural candidate for closer investigation. We also include the intent to specialise, since this seems to be an important part of the motivation in attempting a career in medicine. There were very few observations of the intent to specialise apart from specialisations in medicine and public health; hence, these two are the only ones included in the regression analysis. The results when we include the motivation variables are presented in Table 5 under the column entitled "Model 2".

A personal interest in medicine does not turn out to show a significant effect on the willingness to accept a rural job. We saw previously that a personal interest in medicine is a very important motivational factor, but this does not necessarily imply a higher willingness to accept jobs in rural areas. As the biggest hospitals with the most experienced specialists are in urban areas, we could expect those with a personal interest in medicine to prefer urban areas. On the other hand, doctors in rural areas generally come into closer contact with their patients, and due to a staff shortage, they will do more of the tasks reserved for more experienced doctors in the bigger hospitals. Unfortunately, our analysis reveals no clear answer as to which effect is the strongest.

A planned specialisation in medicine seems to have a negative association with accepting a rural posting, i.e. students who are aiming for a particular specialty in medicine are less likely to accept a job in rural areas, though this result is not significant. This may be due to collinearity (see below). However, we find that a plan to specialise in public health makes it significantly less likely that a student would be willing to accept a job in a rural area. Students that plan to go for this type of specialty have a 50 percentage points lower chance of accepting a rural job (significance level of 5%). Most of the training institutions/hospitals are located in urban areas, so it is difficult to pursue a specialty in a rural area. Many clinicians also express a concern about becoming forgotten or needed too much if they accept a rural position, a result that may cause them to lose an opportunity for further education. It is safer to stay in urban areas to be closer to the authorities who decide who receives the opportunity to train for a specialisation.

#### The influence of training institutions

Differences in admission policies among universities and financing possibilities available to individual students could influence the characteristics of students ultimately entering medical school, and hence the probability of a student accepting a rural job might be related to the medical school a student attended. However, we were not able to establish any such relationship in our data.

The last group of explanatory variables which we explore is the group of variables that can give an indication of how the content of medical training affects the willingness to work in rural areas. We have chosen two variables, specifically community health rotation and fieldwork during studies. The results from the regression that includes all variables are reported in Table 5 under the column entitled "Model 3". The correlation between the medical school a student attended and the other regression variables used in this study is shown in Additional file 1.

By adding variables related to training to our regression model, we find one additional significant result (at a level of 5%): Students who have a community health rotation during their training are less likely to accept a job in a rural area than other students. This effect seems to be quite strong, as the likelihood of accepting a job in a rural area decreases by 34 percentage points if the student has taken part in a community health rotation. We can think of two possible reasons for this.

1) Either the students find out that they dislike rural areas in general, e.g. because of bad infrastructure.

2) It may also be that the content and/or organisation of the community health rotation is inadequate.

In addition, students may be poorly prepared for the challenges they meet in the rotation. These topics require further investigation, as there may be potential for improving the recruitment of new doctors to rural areas with a better organisation of their rural exposure during training. Most likely, the training institutions have a natural and important role to play here.

In their review on the predictors of recruitment and retention, McDonald et al. conclude that a rural background stands out as the primary predictor of entering into rural practice. Nevertheless, it also turns out that the link between a rural placement in training and the later working in a rural practice is more tenuous, although there appears to be an association [21]. Our findings support this: to have parents residing in a rural area is without a doubt the largest influence on a medical student's willingness to accept a job in a rural area of the United Republic of Tanzania. Since the group of students who do not participate in a community health rotation may be a select group, we, like McDonald et al., are not able to establish a causal relationship between the training and the willingness to work in rural areas.

#### Predicted probabilities of accepting a rural job

As we discover in Table 5, the predicted probability of accepting a rural job are 61.4%, 61.6% and 62.9% in Models 1-3, respectively. These probabilities seem unnaturally high, and it is doubtful that we would see the geographical imbalance that we observe if these probabilities were fully representative. We therefore find it important to note that the predicted probabilities of accepting a job in a rural area as provided by the data in this study can and should be thought of as 'upper level' estimates. These estimates are likely to be somewhat biased towards the knowledge that doctors are desperately needed in rural areas and that it would be a 'good thing to do' to go there (see discussion in the methods section of this paper), leading to a higher probability of accepting a rural job than if the answers were not biased. However, even though we may not be able to trust the absolute estimates of this probability, the odds are high that we can trust the probability of not accepting a job in a rural area as being at least 37.1%, as there is little positive bias we can think of when it comes to this measure. Consequently, the estimated probabilities yield an upper level probability that is important to bear in mind when interpreting results and considering policies in addressing the problem of doctor scarcity in rural areas. Furthermore, the relative influence that various characteristics have on the probability of accepting a rural job is not affected by the previously mentioned bias. In spite of the fact that we must assume that the absolute probability of accepting a job in a rural area is somewhat upwardly biased, the analysis still provides valuable information in regard to which characteristics are most likely to affect this probability.

#### General comments to the regression analysis

In order to check for multicollinearity, a Table 6 shows correlations among the variables included in the regression analysis. There seems to be few problems with multicollinearity in our regression model because generally speaking the correlations are relatively low. However, there are a few exceptions: having a rural background is positively correlated with having parents living in a rural area (0.682); planning a specialisation in medicine is negatively correlated with planning a specialisation in public health (0.864); and having done community service during studies is positively correlated with having conducted fieldwork during studies (0.755).

**Table 6:** Correlations between the explanatory variables

	Male student	>26 years	Number of deps	**Urban backgr**.	**Rural backgr**.	Schooling in rural area	Parents live in rural area	Motivated by interest in medicine	Spec. in medicine	Spec. in pub. health	Community health service during studies	Field work during studies
Male student	1											
>26 years	-0.205	1										
Number of dependants	0.116	-0.040	1									
Urban background	0.005	0.085	0.029	1								
Rural background	-0.156	0.172	-0.196	-0.502	1							
Schooling in rural area	-0.253	0.073	-0.140	-0.073	0.378	1						
Parents live in rural area	-0.174	-0.056	-0.131	-0.393	0.682	0.505	1					
Motivated by interest in medicine	0.217	0.007	-0.141	-0.134	0.049	0.097	0.036	1				
Specialisation in medicine	0.092	-0.099	0.174	-0.101	-0.037	-0.058	-0.060	-0.09	1			
Specialisation in public health	-0.014	0.171	-0.155	0.154	0.014	-0.086	0.034	-0.08	-0.864	1		
Community health service during studies	0.097	-0.188	0.120	0.036	-0.240	0.170	-0.213	0.06	0.070	-0.14	1	
Fieldwork during studies	0.067	-0.109	0.099	-0.052	-0.121	0.186	-0.089	0.02	0.100	-0.17	0.755	1

In order to avoid multicollinearity, an alternative could be to exclude one of the correlated variables. However, there may be several problems with such an approach. When dropping one variable out of the analysis, we may create an unintended bias in the estimates [31]. Moreover, and possibly more importantly for our policy-oriented analysis, there is the risk of excluding variables for which there is good reason to think are important, in order to understand the phenomenon of interest and its implications for policy. Even though having parents in a rural area and having a rural background are correlated more than we would desire from a statistical analysis perspective, they capture quite different links to a rural area, thus yielding very different implications for policy making. This same argument goes for the two included specialties; they represent very different directions in specialisation, and may tell us something about the types of doctors willing to work in rural areas. Similarly, fieldwork and community health service are two different ways of exposing students to rural health issues. Even though they may capture some of the same effect, they give different policy implications for the training of future doctors. For all three pairs of correlations, we see that only one of the two correlated variables has a significant effect on the willingness to accept a rural position. However, those variables that turn out to be significant work in directions that fit well with findings from other studies, making it reasonable to believe that we are actually capturing some interesting and real relationships. An increased sample size would be the best way to improve the study with respect to the problems of collinearity. However, this was not possible in our case. It should also be noted that collinearity in some of the variables does not exert an influence on the coefficient of any other variable.

Bearing in mind these considerations, it is reassuring to note that our results are quite stable concerning various model specifications. The variables that were significant in the first model were also significant at the same level, and with similar marginal effects, in both the second and third models. As we can read from the last rows in Table 5 the goodness of the fit increased as the initial model was expanded, hence Model 3 had the best fit with the data.

## Conclusions

The above-mentioned predictors for the willingness to participate in rural practice suggest that it may be time to re-examine the admission policies of Tanzanian medical schools. Our results show that policies should be considered that aim at selecting the correct students (a fair representation of students with a rural upbringing and the 'right' specialty preference), and exposing them to the curriculum and positive experiences needed in order to become motivated for and to succeed in primary care in a rural setting.

Our analysis also demonstrated that many students are joining medical school without a primary interest in medicine, and that over two-thirds are less motivated for a medical career on completion of medical school than they were at the time of entry. Such results certainly give rise for concern.

Medical schools may perpetuate the imbalance in the availability of human resources for health in the United Republic of Tanzania through an unintended bias in the selection of candidates for training (thus, the wrong students). This imbalance is also perpetuated by training programs that do not seem to adequately prepare a new doctor for rural health care challenges, i.e. a clinical curriculum that to some extent is rural-unfriendly (hence, the wrong schools).

Finally, we have some questions which seem to be highly relevant after having examined all the evidence that exists in this area, including ours. We do not aim to answer these questions here, but feel confident that they offer interesting and necessary research areas.

1. Is academic performance in high school a good enough criterion for selecting candidates to attend medical school? Many arbitrary factors affect this criterion (including the differences in educational opportunities for rural and urban dwellers). As a consequence, "rural friendly" students who are intellectually fit to be doctors are left out.

2. How and where do Tanzanian universities go wrong in developing training programs that ultimately leave their students demotivated?

3. Have the medical training institutions failed to meet the training needs of "clinically-oriented" students and interns?

4. What potential impact may that have on the availability of doctors willing to work in the rural areas of the United Republic of Tanzania?

## Competing interests

The authors declare that they have no competing interests.

## Authors' contributions

BKL developed the study protocol, collected the data and performed the preliminary analysis. JRK has been responsible for the econometric analysis and the discussion of the results. Both authors have taken part in the general discussion, and both authors have agreed that their work can be published.

## Supplementary Material

Additional file 1 **Correlation between the school dummies and the other regression variables**. The file contains data in a tabular form, demonstrating the correlation between attending a particular medical school and the regression variables tested in the study. The variables were male gender, age above 26 years, rural/urban backgrounds, schooling in a rural area, having parents living in a rural area, motivation by an interest in medicine, intended specialization in medicine, intended specialization in public health, community service during studies, and having done field work during studies. Click here for file

## References

[B1] Munga M Mæstad O Measuring inequalities in the distribution of health workers: the case of Tanzania Human Resources for Health 2009 7 4 10.1186/1478-4491-7-4 PMC2655278 19159443

[B2] WHO World health report 2000 - Health systems: improving performance Geneva 2000

[B3] Chen L Evans T Anand S Boufford JI Brown H Chowdhury M Cueto M Dare L Dussault G Elzinga G Human resources for health: overcoming the crisis Lancet 2004 364 1984 1990 10.1016/S0140-6736(04)17482-5 15567015

[B4] WHO World Health Report 2006 - Working together for health Geneva 2006

[B5] Huddart J Picazo OA The health sector human resource crisis in Africa: an issues paper 2003 Washington, D.C.: Academy for Educational Development [AED], Support for Analysis and Research in Africa [SARA]

[B6] Khan MM Hotchkiss DR Berutti AA Hutchinson PL Geographical aspects of poverty and health in Tanzania: Does living in a poor area matter? Partners for health reform plus 2003 Tulane University

[B7] Anand S Barnighausen T Human resources and health outcomes: cross-country econometric study Lancet 2004 364 1603 1609 10.1016/S0140-6736(04)17313-3 15519630

[B8] Nullis-Kapp C Health worker shortage could derail development goals Bulletin of the World Health Organisation 2005 83 5 6 PMC2623463 15682240

[B9] Dominick A Kurowski C Human resources for health: an appraisal of the status quo in Tanzania mainland 2005 Ifakara health research and development centre & The World Bank

[B10] Kurowski C Wyss K Abdulla S Yémadji N Mills A Human resources for health: Requirements and availability in the context of scaling-up priority interventions in low-income countries: Case studies from Tanzania and Chad DID Reports: Department for International Development 2004

[B11] World Bank World Bank Development Indicators 2001 Washington 2001

[B12] WHO estimates of health personnel 1998 WHO, Geneva

[B13] University of Dar es Salaam Tracer Studies in a Quest for Academic Excellency 2004 Dar es Salaam University Press

[B14] SADC Human development report 2000 2001 SAPES. Harare

[B15] World Bank World Bank Development Indicators 2002 Washington 2002

[B16] O'Shea A Rawls A Golden E Cecil R Slota E Biezychudek K Action Now on the Tanzanian Health Workforce Crisis: Expanding Health Worker training-The Twiga initiative 2009 Touch Foundation

[B17] Talley RC Graduate medical education and rural health care Academic Medicine 1990 65 22 25 10.1097/00001888-199012000-00029 2252512

[B18] Rabinowitz HK Diamond J Hojat M Hazelwood CE Demographic, educational and economic factors related to recruitment and retention of physicians in rural Pennsylvania The journal of rural health 1999 15 212 218 10.1111/j.1748-0361.1999.tb00742.x 10511758

[B19] Somers G Do rural mentors, undergraduate clubs and rural rotations increase the medical students' intention to practice in the country? Scientific Forum 2002 2002 Australian Centre for Rural and remote medicine

[B20] Azer S Simmons D Elliot S Rural training and the state of rural health services: effect of rural background on the perception and attitude of first year students at The University of Melbourne Australian Journal of Rural Health 2001 9 178 10.1046/j.1038-5282.2001.00359.x 11488702

[B21] McDonald J Bibby L Caroll S Recruiting and retaining general practitioners in rural areas: improving outcomes through evidence-based research and community capacity building. Centre for health research and practice 2002 University of Ballarat

[B22] Leon B Maldistribution of the medical workforce in Tanzania: Predictors of willingness for rural medical practice among Tanzanian final year medical students 2005 University of Dar es Salaam

[B23] Dussault G Franceschini MC Not enough there, too many here: understanding geographical imbalances in the distribution of the health workforce Human Resources for Health 2006 4 12 10.1186/1478-4491-4-12 PMC1481612 16729892

[B24] Brooks RG Walsh M Mardon RE Lewis M Clawson A The roles of nature and nurture in the recruitment and retention of primary care physicians in rural areas: a review of the literature Academic Medicine 2002 77 790 798 10.1097/00001888-200208000-00008 12176692

[B25] Wibulpolprasert S Inequitable distribution of doctors: can it be solved? Human Resources Development Journal 1999 3 1 45

[B26] Kamien M Buttfield IH Some solutions to the shortage of general practitioners in rural Australia. Part 1: Medical school selection Medical Journal of Australia 1990 153 105 107 2366689

[B27] Serneels P Lindelow M Garcia-Montalvo J Barr A For Public Service or Money: Understanding Geographical Imbalances in the Health Workforce 2005 The World Bank 10.1093/heapol/czm005 17463013

[B28] Chomitz KM Setiadi G Azwar A Ismail N Widiyarti What Do Doctors Want? Developing Incentives for Doctors to Serve in Indonesia's Rural and Remote Areas 1998 The World Bank

[B29] Standing H Gender - a Missing Dimension in Human Resource Policy and Planning for Health Reforms Human Resources Development Journal 2000 4 1 27 42

[B30] King MH Primary surgery volume one (Non-trauma) 1990 Oxford University Press

[B31] Wooldridge JM Introductory Econometrics: A Modern Approach 2006 3 Thomson South-Western

