# Supplementary material for: Wrong schools or wrong students? The potential role of medical education in regional imbalances of the health workforce in the United Republic of Tanzania
Source: Hum Resour Health. 2010 Feb 26;8:3. doi: 10.1186/1478-4491-8-3 (PMC2837610; doi:10.1186/1478-4491-8-3)
Supplement: Additional file 1 — Correlation between the school dummies and the other regression variables. The file contains data in a tabular form, demonstrating the correlation between attending a particular medical school and the regression variables tested in the study. The variables were male gender, age above 26 years, rural/urban backgrounds, schooling in a rural area, having parents living in a rural area, motivation by an interest in medicine, intended specialization in medicine, intended specialization in public health, community service during studies, and having done field work during studies. [file 1478-4491-8-3-S1.DOC]

**Correlation between the school dummies and the other regression variables**

|  | **Male student** | **>26 years** | **Number of dependants** | **Urban background** | **Rural background** | **Schooling in rural area** | **Parents live in rural area** | **Motivated by interest in medicine** | **Specialisation in medicine** | **Specialisation in public health** | **Community health service during studies** | **Field work during studies** |
| --- | --- | --- | --- | --- | --- | --- | --- | --- | --- | --- | --- | --- |
| HKMU | 0.279 | -0.192 | 0.105 | 0.083 | -0.098 | -0.151 | -0.024 | -0.048 | 0.113 | -0.056 | 0.112 | 0.112 |
| KCMC | 0.209 | -0.040 | -0.012 | -0.009 | 0.023 | 0.001 | 0.087 | 0.221 | 0.125 | -0.102 | 0.253 | 0.253 |
| MUCHS | -0.353 | 0.16 | -0.060 | -0.048 | 0.047 | 0.100 | -0.054 | -0.146 | -0.182 | 0.120 | 0.160 | 0.160 |
